# Enhanced Electrochemical Catalytic Efficiencies of Electrochemically Deposited Platinum Nanocubes as a Counter Electrode for Dye-Sensitized Solar Cells

**DOI:** 10.1186/s11671-015-1177-8

**Published:** 2015-12-02

**Authors:** Yu-Hsuan Wei, Ming-Chi Tsai, Chen-Chi M. Ma, Hsuan-Chung Wu, Fan-Gang Tseng, Chuen-Horng Tsai, Chien-Kuo Hsieh

**Affiliations:** Department of Engineering and System Science, National Tsing Hua University, Hsinchu, 30013 Taiwan, Republic of China; Department of Chemical Engineering, National Tsing Hua University, Hsinchu, 30013 Taiwan, Republic of China; Department of Materials Engineering, Ming Chi University of Technology, New Taipei City, 24301 Taiwan, Republic of China

**Keywords:** Dye-sensitized solar cells, Platinum nanocubes, Electrochemical deposition, Counter electrode

## Abstract

Platinum nanocubes (PtNCs) were deposited onto a fluorine-doped tin oxide glass by electrochemical deposition (ECD) method and utilized as a counter electrode (CE) for dye-sensitized solar cells (DSSCs). In this study, we controlled the growth of the crystalline plane to synthesize the single-crystal PtNCs at room temperature. The morphologies and crystalline nanostructure of the ECD PtNCs were examined by field emission scanning electron microscopy and high-resolution transmission electron microscopy. The surface roughness of the ECD PtNCs was examined by atomic force microscopy. The electrochemical properties of the ECD PtNCs were analyzed by cyclic voltammetry, Tafel polarization, and electrochemical impedance spectra. The Pt loading was examined by inductively coupled plasma mass spectrometry. The DSSCs were assembled via an N719 dye-sensitized titanium dioxide working electrode, an iodine-based electrolyte, and a CE. The photoelectric conversion efficiency (PCE) of the DSSCs with the ECD PtNC CE was examined under the illumination of AM 1.5 (100 mWcm^−2^). The PtNCs in this study presented a single-crystal nanostructure that can raise the electron mobility to let up the charge-transfer impedance and promote the charge-transfer rate. In this work, the electrocatalytic mass activity (MA) of the Pt film and PtNCs was 1.508 and 4.088 mAmg^−1^, respectively, and the MA of PtNCs was 2.71 times than that of the Pt film. The DSSCs with the pulse-ECD PtNC CE showed a PCE of 6.48 %, which is higher than the cell using the conventional Pt film CE (a PCE of 6.18 %). In contrast to the conventional Pt film CE which is fabricated by electron beam evaporation method, our pulse-ECD PtNCs maximized the Pt catalytic properties as a CE in DSSCs. The results demonstrated that the PtNCs played a good catalyst for iodide/triiodide redox couple reactions in the DSSCs and provided a potential strategy for electrochemical catalytic applications.

## Background

In the recent years, Grätzel and many scientists have been attracted to study potential candidates of dye-sensitized solar cells (DSSCs) for next-generation solar cells [1–3]. DSSCs have many outstanding features such as the highlight of good photoelectric conversion efficiency (PCE) and the potential for its low cost and simple fabrication. The DSSCs have three major parts to assemble the sandwich structure, including the working electrode (WE), the electrolyte, and the counter electrode (CE). First of all, the WE is composed of porous nanocrystal titanium dioxide (TiO_2_) nanoparticles adsorbed with dye molecules on a conductive glass substrate. Secondly, electrolyte contains iodide/triiodide redox couples (I^−^/I_3_^−^) in an organic solvent between the WE electrode and the CE electrode. Finally, CE is an electrocatalytic activation layer deposited on a conductive glass, which is typically a platinum (Pt) film deposited with a fluorine-doped tin oxide (FTO) glass. Until recently, many scientists focused on the performance improvement of the WE for the efficient sunlight harvesting [4–6]. However, CE also plays an important role in DSSCs; there are three major functions of the CE in DSSCs: (1) transfer electrodes to make the cell as a complete circuit, (2) regenerate the I_3_^−^ in the interface of the electrolyte/CE, and (3) reduce the I^−^/I_3_^−^ redox couples in order to keep low potential to minimize energy losses. The most common nanomaterials applied to the CE for DSSC investigations were Pt [7, 8], carbon nanotubes [9, 10], graphene [11, 12], graphene oxide [13], molybdenum sulfide [14–16], etc. Among those electrocatalytic materials, Pt exhibits the best electrocatalytic performance, which was widely used in fuel cells, biosensors, and chemical sensor development of the advanced technologies for kinds of the environmental, renewable energy, and industrial process [17–20]. In the DSSCs, Pt was one of the best materials to enhance the performance for CE due to its functional electrocatalytic activity for the I^−^/I_3_^−^ couple redox reactions, and it also performs the high conductivity property [21, 22]. The investigations of preparations for kinds of Pt morphologies and nanostructures as a CE have attracted much attention for the improvement of DSSC’s efficiency [23–26]. It has been found that Pt has distinct electrocatalytic activity on specific crystalline planes for the electrochemical applications [25], which suggests that the maximum electrocatalytic activity may be achieved by controlling the single crystallinity in fabricating the Pt nanomaterials. However, the challenge to synthesize the unique morphology of single-crystal Pt nanomaterial still remains [27–29].

It is well known that the catalytic activity of nanocrystals is strongly related to their surface structure because the electrochemical reactions are surface structure sensitive. Pt is an expensive and a precious metallic element owing to its scarcity, and hence, the preparations of Pt nanocatalysts with enhanced electrocatalytic activity and utilization efficiency have been an important research focus. Very recently, intense attention has been paid to the synthesis and the analysis of Pt nanocubes (PtNCs) [30, 31]. In contrast to the traditional Pt film deposition from expensive sputtering vacuum equipment, electrochemical deposition (ECD) was found to be an efficient method at room temperature and a simple and low-cost process to deposit Pt nanostructure [32–35]. In this study, we described our investigation of the PtNCs, which was directly deposited onto the fluorine-doped SnO_2_ conducting glass (FTO, 8 Ω/sq., 2.2 mm in thickness, TEC-7, Hartford) by a novel pulse-mode ECD (pulse-ECD) method. The surface morphology and nanostructure of the prepared pulse-ECD PtNCs were examined by field emission scanning electron microscopy (FESEM) and high-resolution transmission electron microscopy (HRTEM). The surface roughness of the prepared samples was examined by atomic force microscopy (AFM). Pt loading was examined by inductively coupled plasma mass spectrometry (ICP-MS). The electrocatalytic performances were considered by cyclic voltammetry (CV), Tafel polarization, and electrochemical impedance spectrum (EIS). It was found that the influence of PtNCs with the single-crystal nanostructure was significant on the resulting electroactivities and therefore enhanced the performance of DSSCs. The PCE of the DSSCs with the pulse-ECD PtNCs on FTO as a CE was examined under the illumination of AM 1.5 (100 mWcm^−2^), which was revealed to exhibit an excellent PCE of 6.48 %, higher than the cells using the conventional Pt film CE (6.18 %).

## Methods

### Preparation of the TiO_2_ Working Electrodes

A FTO conducting glass was firstly cleaned with deionized (DI) water, acetone, and isopropyl alcohol (IPA, 99.5 %, Sigma-Aldrich), sequentially. The nanocrystalline TiO_2_ nanoparticles were used to coat as a thin film, two kinds of TiO_2_ nanoparticle layers were coated on the FTO glass by using the print-screen method, and the area of TiO_2_ nanoparticle layer was 0.049 cm^2^. The first layer (10 μm in thickness) served as the interlayer on which a layer of light-scattering anatase TiO_2_ particles (2 μm in thickness) was coated. The TiO_2_-coated WE was dried at 120 °C, then gradually heated to 550 °C for 30 min in ambient air, and then cooled slowly to room temperature. After calcination, the TiO_2_-coated WE was immersed in a N719 dye (Solaronix) solution (0.3 mM in a mixture of acetonitrile and tert-butyl alcohol) for 24 h at room temperature. The dye-adsorbed TiO_2_-coated WE was washed with acetonitrile (ACN) and dried at room temperature for a few seconds to remove the remaining dye.

### Preparation of the Pt Counter Electrodes

There were two different methods used to prepare the Pt CEs in this study. Method I was the conventional Pt film prepared by electron beam evaporation and used as a reference electrode. The Pt film was deposited on the FTO glass to a thickness of 50 nm in order to ensure high PCE of the reference Pt film CE [36]. Method II was the pulse-ECD PtNCs, where the FTO glass was subjected to a pulse-ECD method in a plating bath containing an Ar-saturated Pt precursor aqueous solution (0.2 mM H_2_PtCl_6_˙6H_2_O and 0.1 M H_2_SO_4_ in DI water) at a controlled temperature of 30 °C under ambient pressure. In each scan cycle, voltages of 0 and −0.45 V_SCE_ were each applied for the duration of 1 s, and 500 scans were used to deposit the PtNCs on the FTO glass. The pulse-ECD PtNCs were performed using a potentiostat/galvanostat (PGSAT 302N, Autolab, EcoChemie) in a conventional three-electrode cell, and a Pt plate and a saturated calomel electrode were used as the CE and the reference electrode, respectively.

### Characterization of the Pulse-ECD PtNC Electrodes

The surface morphology and the nanostructure of the pulse-ECD PtNCs were characterized by using FESEM (JEOL, JSM-6330F) and HRTEM (JEOL-2100F), respectively. The AFM (Park system, XE-70™) with a nanoscope IV controller by Digital Instruments Inc. was carried out to examine the surface roughness in an ambient environment. CV was used to examine the redox activities of the prepared CE samples in a three-electrode configuration, at a scan rate of 20 mVs^−1^ at room temperature. The solution used for CV measurements contained 1 mM I_2_, 10 mM LiI, and 0.1 M LiClO_4_ in ACN solution. The EIS was applied to study the charge-transfer properties of the CE. During the scan for the EIS, the frequency ranged from 10^5^ to 10^−1^ Hz and used an applied electric bias potential of 10 mV. Nyquist plots were held and examined using the aforementioned potentiostat/galvanostat equivalent circuit model with Autolab FRA software. The Tafel measurements were used to calculate the exchange current density. Tafel polarization curves were performed with a scanning rate of 1 mV s^−1^ in the potential range of 0.1 to −0.1 V. The symmetric dummy cells were used for both EIS and Tafel measurements, and both EIS and Tafel measurements were analyzed using a potentiostat/galvanostat (PGSAT 302N, v4.9 Autolab, EcoChemie B.V.). The Pt loadings of the prepared samples were measured with the ICP-MS (SCIEX ELAN 5000, Perkin Elmer).

### DSSC Assembly and Photovoltaic Performance Measurement

In order to assemble the DSSCs, a 60-μm-thick hot melt spacer (SX1170-60, Solaronix) was sandwiched between the WE and CE by heating at 100 °C for a few seconds. The liquid, iodide-based electrolyte (AN-50, Solaronix) was injected into the space between the WE and CE of the DSSCs. The DSSC devices were illuminated by a grade A quality solar simulator with a light intensity of 100 mWcm^−2^ (AM 1.5) to measure the photocurrent–voltage curves, and the solar light was calibrated with a standard silicon cell (calibrated at NREL, PVM-81).

## Results and Discussion

### Characterization of the Pt Counter Electrodes

**Fig. 1:**
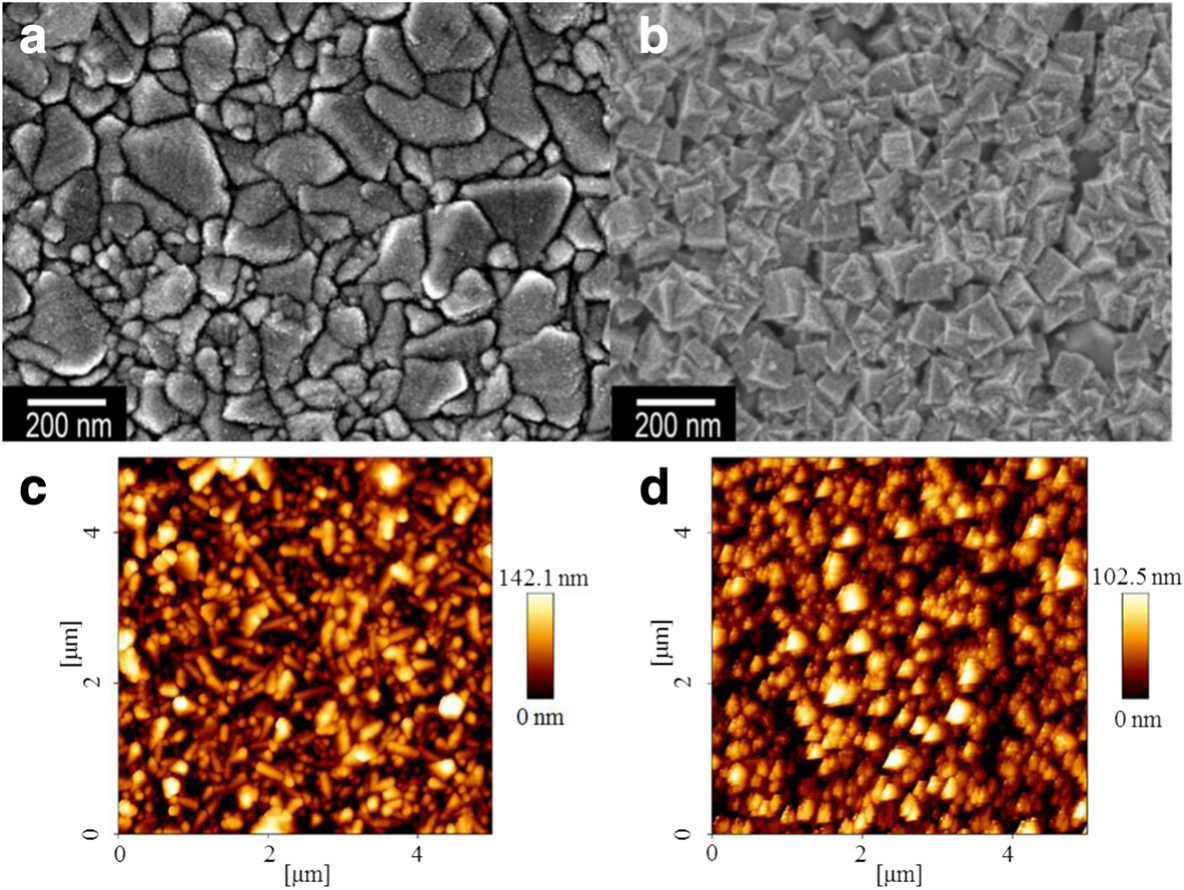
FESEM images of **a** the Pt film on the FTO glass and **b** pulse-ECD PtNCs on the FTO glass. AFM topographic images of **c** the Pt film and **d** pulse-ECD PtNCs

**Fig. 2:**
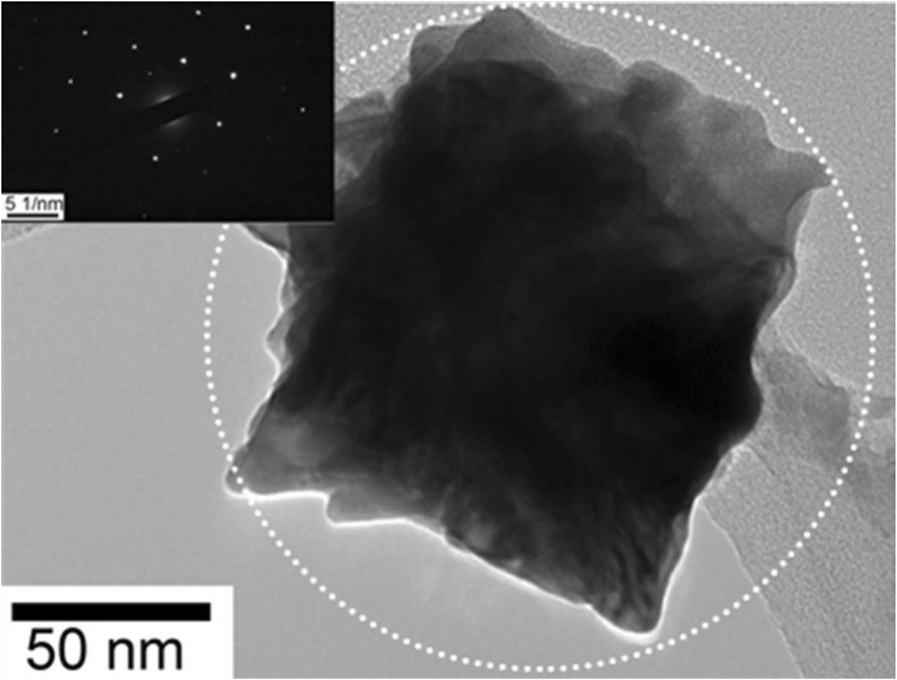
HRTEM micrograph of the pulse-ECD PtNC. *Insert* is the SAED pattern

Figure 1a, b shows the FESEM images of the Pt film and pulse-ECD PtNCs, respectively. The prepared electron beam evaporation deposited 50 nm Pt on the FTO surface sample as shown in Fig. 1a. Figure 1b shows the prepared pulse-ECD PtNC sample with highly uniform film on the FTO surface that exhibited the PtNC morphology in a cubic shape, and the side of the PtNCs was around 100 nm. Figure 1c, d shows the AFM topographic images of the Pt film and pulse-ECD PtNCs, respectively. Table 1 shows the surface roughness (average roughness (Ra) and RMS roughness (Rq)) for the Pt film and PtNCs, respectively. From the AFM results, both Ra and Rq of the PtNCs were higher than those of the Pt film, and this phenomenon coincided with the FESEM images. And, the higher roughness of surface enhanced the electrochemical catalytic performance of the I_3_^−^ reduction activity. Figure 2 shows the HRTEM micrograph of the PtNC, and the related selected-area electron diffraction (SAED) pattern of the PtNC was also shown as the insert. From the HRTEM micrograph, firstly, we could clarify the single facet size of the PtNCs. As shown in Fig. 2, one whole facet of the PtNC was captured, and the side length of a nanocube could be measured as about 100 nm, which was consistent with the estimation from the SEM analysis above. Secondly, for revealing the crystallinity and detailed structure of the Pt nanocube, SAED analysis was performed on the whole nanocube by modifying the aperture. As shown in the insert in Fig. 2, there existed only a single set of diffraction pattern, which indicated that the whole Pt nanocube presented the single-crystal characteristic. Further revealing the feature of this set of diffraction pattern, the classic diffraction of Face-Centered Cubic (FCC) structure with [001] zone axis was confirmed by sorting basic diffraction reference [37]. Besides, the d-spacing (*d*) of the crystal was also calculated by Bragg’s equation: *d* = *λI*/*r*, where the *λ* is the wavelength of electron, *I* is the camera focal length, and *r* is the distance between the central spot and the diffraction spot. The d-spacing of the object was then calculated as 1.9 Å along the [200] direction, which was approximate to the theoretical value of 1.965 Å of Pt. According to these results from HRTEM, the single-crystal crystallinity of a 100-nm-side-length nanocube was revealed. Through the diffraction pattern and d-spacing calculation, the FCC structure and 1.9-Å d-spacing indirectly indicated that the nanocube was consisted of Pt with minimum defects due to the clear pattern without any amorphous rings. Combining the result of SEM analysis, the uniform, continuous nanocubical film with single-crystalline facet feature was achieved by pulse-ECD approach. We suggested that the PtNC film provided low recombination of transport carriers due to its high-quality single crystallinity, and no more grain boundaries existed in a PtNC to hinder the current flow. Additionally, the performance of catalytical function could be promoted due to its high specific surface area feature, and the rise of PCE was expected in the DSSC application. From the ICP-MS measurements, the Pt loading values of the PtNCs and Pt film were 0.203 and 0.378 mgcm^−2^, respectively, which were summarized in Table 2.

**Table 1:** AFM roughness values of the Pt film and pulse-ECD PtNCs

Sample	Average roughness (Ra; nm)	RMS roughness (Rq; nm)
Pt film	20	24
PtNCs	30	37

**Table 2:** Summary of the electrochemical characteristics and Pt loadings of the reference Pt film CE and the pulse-ECD PtNC CE

CE	*I*_pa_ (mAcm^−2^)	*I*_pc_ (mAcm^−2^)	*R*_s_ (Ω)	*R*_ct_ (Ω)	*Z*_N_ (Ω)	*J*_lim_ (mAcm^−2^)	Pt_Loading_ (mgcm^−2^)
Pt film	0.94	−0.57	23.5	78.8	4.83	22	0.378
PtNCs	1.11	−0.83	25.2	32.4	4.04	34	0.203

### Electrochemical Properties of the Pt Counter Electrodes

**Fig. 3:**
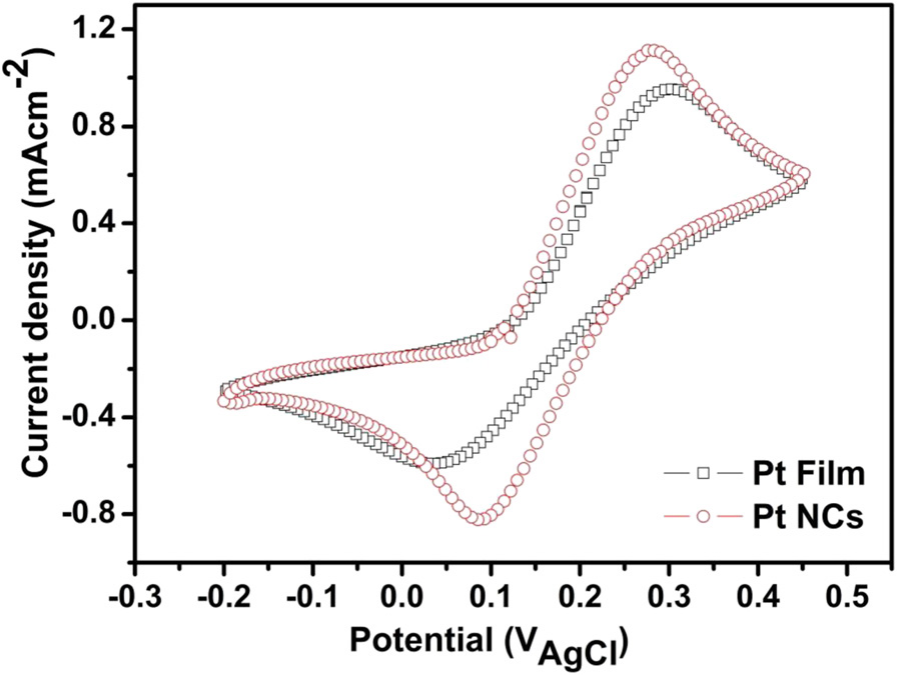
CV curves of the Pt film CE and pulse-ECD PtNC CE

To investigate the reaction kinetics and electrocatalytic activities of the pulse-ECD PtNC electrode and to compare it with that of a conventional Pt film electrode for the redox reactions of I^−^/I_3_^−^ couples, we used the CV measurement in order to examine the charge-transfer current density of the reduction and oxidation abilities. Figure 3 shows the CV measurements of the PtNCs and Pt film, respectively. As we can see from Fig. 3, an anodic peak current density (*I*_pa_) and a cathodic peak current density (*I*_pc_) were observed as an oxidation peak on the right side and a reduction peak on the left side, respectively, which corresponded to the oxidation ability of I^−^ ions and the reduction ability of I_3_^−^ ions, respectively. The peaks can be attributed to the reduction of triiodide according to Eq. (1) [38].

1 $$ {{\mathrm{I}}_3}^{-}+2{e}^{-}\leftrightarrow 3{\mathrm{I}}^{-} $$

According to Fig. 3, the *I*_pc_ of the PtNC electrode was −0.83 mAcm^−2^, and the Pt film electrode was −0.57 mAcm^−2^, which means a higher redox reaction rate in the interface between the PtNC electrode and electrolyte. Our prepared pulse-ECD PtNC electrode demonstrated a superior electrocatalytic activity for the I_3_^−^ ion reduction reactions.

**Fig. 4:**
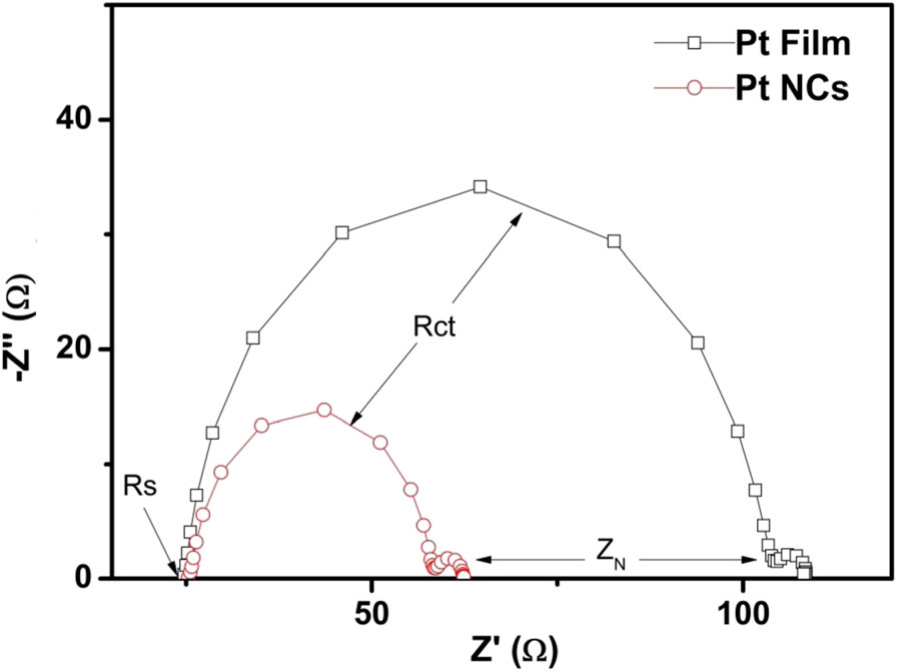
EIS analysis of the pulse-ECD PtNC CE and Pt film CE of the Nyquist plots

EIS analysis was performed with a spacer sealed symmetric cell consisting of two identical CEs to study the internal resistances and charge-transfer impedance of the various CE samples. Figure 4 shows the Nyquist plots measured from the pulse-ECD PtNC and Pt film symmetrical cells, respectively. The substrate series resistance (*R*_s_), charge-transfer resistance (*R*_ct_), and diffusion impedance (*Z*_N_) were obtained from the Nyquist plot. The first semicircle scanned in the high-frequency region represented the charge-transfer reactions, and the second semicircle scanned in the low-frequency region represented the Nernst diffusion impedance between the electrodes. The *R*_ct_ values of the PtNCs and Pt film were 32.4 and 78.8 Ω, respectively, and the pulse-ECD PtNCs showed the lowest *R*_ct_ value of 32.4 Ω.

**Fig. 5:**
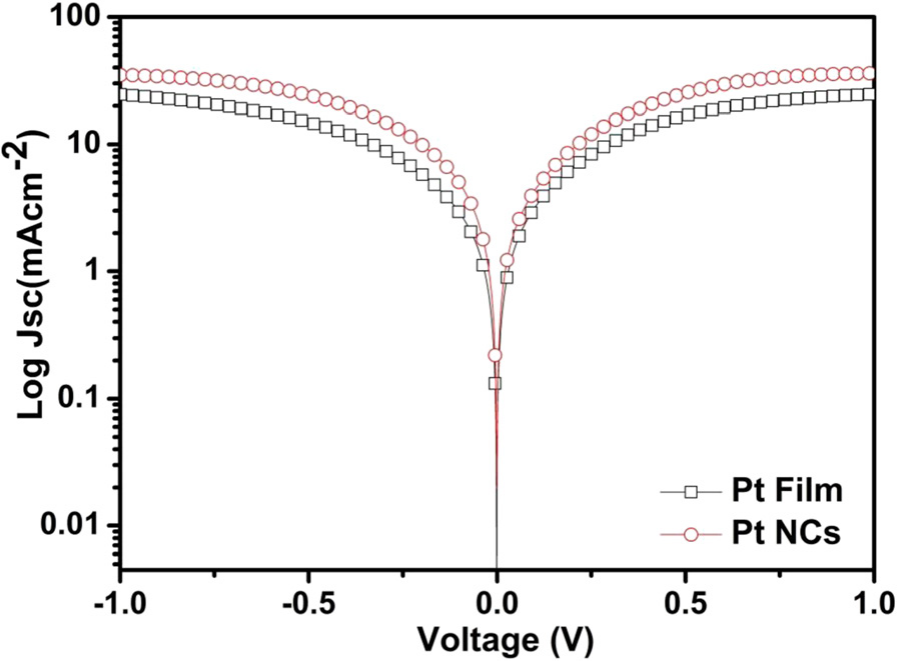
Tafel curves of the pulse-ECD PtNC CE and Pt film CE

Figure 5 shows the Tafel polarization curves of the symmetrical cells based on the pulse-ECD PtNC and Pt film electrodes, respectively. As we can see from Fig. 5, the curves showed logarithmic current density (log *J*) as voltage (*V*). The exchange current density (*J*_0_) and the limiting diffusion current density (*J*_lim_) could be obtained from these curves, which could be used to point out the electrocatalytic activities of the CEs. Comparing with the Pt film CE, the pulse-ECD PtNCs performed a larger exchange current density (*J*_0_), which meant that the PtNCs presented higher electrocatalytic activity and lower charge-transfer resistance at the electrolyte–electrode interface. *J*_0_ value was inversely related with *R*_ct_ as according to Eq. (2) [39]

2 $$ {J}_0=\frac{RT}{nF{R}_{\mathrm{ct}}} $$

where *R*, *T*, *n*, and *F* represented the gas constant, the temperature, the number of electrons transferred in the reduction reaction, and the Faraday constant, respectively.

As shown in Fig. 5, the limiting current density (*J*_lim_) of the PtNCs and Pt film were 34 and 22 mAcm^−2^, respectively. It could be observed that the pulse-ECD PtNC CE owned a larger *J*_lim_ value of 34 mAcm^−2^. The PtNC CE displayed an enhanced *J*_lim_, which was in good agreement with the EIS values in terms of Eq. (2) and the lower *R*_ct_ of the PtNCs. The *J*_lim_ also depended on the diffusion coefficient of the I^−^/I_3_^−^ redox couples in the DSSCs according to Eq. (3) [16].

3 $$ D=\frac{l}{2nFC}{J}_{\lim } $$

According to Eq. (3), *D*, *l*, *n*, *F*, and *C* represented the diffusion coefficient of the triiodide, the thickness of spacer, the number of electrons involved in the reduction of triiodide at the electrode, the Faraday constant, and the concentration of triiodide, respectively.

Table 2 summarizes the values of *I*_pa_, *I*_pc_, *R*_s_, *R*_ct_, *Z*_N_, *J*_lim_, and Pt loading from the electrochemical measurement results obtained from the CV curves, Nyquist plots, Tafel polarization curves, and ICP-MS measurements. As shown in Table 1, comparing the PtNCs and Pt film, our pulse-ECD PtNC CE provided a better *I*_pc_ of −0.83 mAcm^−2^, a lower *R*_ct_ of 32.4 Ω, and a higher *J*_lim_ of 34 mAcm^−2^. Meanwhile, our prepared pulse-ECD PtNC CE performed the superior electrocatalytic properties.

On the other hand, the result of the CV curves displayed that the electroactivity of PtNCs with (100) plane was superior to the Pt film in triiodide reduction reaction (*I*_pc_ obtained from CV). The mass activity (MA) was widely used to examine the Pt electrocatalytic activity for the electrochemical DMFC (direct methanol fuel cell) [40–42]. The MA can be calculated from Eq. (4) [41].

4 $$ \boldsymbol{M}\boldsymbol{A} = \boldsymbol{c}\boldsymbol{urrent}\ \boldsymbol{density}/\ \boldsymbol{P}\boldsymbol{t}\ \boldsymbol{loading}\ \boldsymbol{per}\ \boldsymbol{c}{\boldsymbol{m}}^{\boldsymbol{2}} $$

According to Eq. (4), we herein utilized the MA calculation to examine the Pt electrocatalytic activity for the triiodide reduction reaction in DSSCs. Table 3 shows the calculation of MA, and the MA of the Pt film and PtNCs was 1.508 and 4.088 mAmg^−1^, respectively. As shown in Table 3, comparing the PtNCs and Pt film, the MA of the PtNCs was 2.71 times than that of the Pt film. PtNCs showed the outstanding utilization efficiency.

**Table 3:** The MA values of the Pt film and pulse-ECD PtNCs

Sample	*I*_pc_ (mAcm^−2^)	Pt _Loading_ (mgcm^−2^)	MA (mAmg^−1^)
Pt film	−0.57	0.378	1.508
PtNCs	−0.83	0.203	4.088

The pulse-ECD PtNCs not only owned the unique single-crystal nanostructure but also decreased the Pt loading as shown in Table 2. Pulse-ECD PtNCs still kept the superior electrocatalytic properties and made a better Pt utilization efficiency as the CE for DSSCs as shown in Table 3. We herein suggested that our prepared specific crystallinity of PtNCs with a unique cubic shape exposed the low-index facets to enhance the electrocatalytic activities at the electrolyte–electrode interface; it might be that the single-crystal structure of PtNCs reduced the internal defects which led to the decrease of the electron loss pathway and enhance the catalytic efficiency. Compared with the conventional Pt film, the pulse-ECD PtNCs not only decreased the Pt loading but also held the superior electrocatalytic properties.

### Characterization of the DSSCs

The photovoltaic characteristics of the DSSCs were executed by measuring the photocurrent–photovoltage (*J*–*V*) characteristic curve under the simulated solar illumination of 100 mWcm^−2^ using a solar simulator (AM 1.5, Oriel 91160) in an ambient atmosphere. The photovoltaic characteristics of the DSSCs were open-circuit voltage (*V*_oc_), short-circuit photocurrent density (*J*_sc_)_,_ fill factor (FF), and PCE (*η* %). The DSSC’s FF could be estimated from Eq. (5) [16].

5 $$ \mathrm{F}\mathrm{F}=\frac{V_{\max}\times {J}_{\max }}{V_{\mathrm{oc}}\times {J}_{\mathrm{sc}}} $$

The DSSC’s PCE can be calculated from Eq. (6) [16].

6 $$ \eta \left(\%\right)=\frac{V_{\mathrm{oc}}\times {J}_{\mathrm{sc}}\times \mathrm{F}\mathrm{F}}{P_{\mathrm{in}}}\times 100 $$

**Fig. 6:**
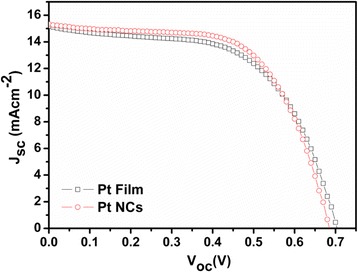
Photocurrent–voltage curves of DSSCs composed of the pulse-ECD PtNC CE and Pt film CE

The *J*–*V* curves of the DSSCs with the pulse-ECD PtNC CE and the Pt film CE were shown in Fig. 6, and the photovoltaic characteristic parameters were summarized in Table 4. The *J*_sc_, *V*_oc_, and FF of the DSSCs with a reference Pt film CE were 15.18 mAcm^−2^, 0.7 V, and 0.58, respectively, which yielded a PCE of 6.18 %. The corresponding parameters obtained from the DSSCs fabricated by the PtNC CE in this work were 15.33 mAcm^−2^, 0.69 V, 0.61, and 6.48 %, respectively. It was worthy to note that the DSSCs with PtNC CE performed higher *I*_pc_ and *J*_lim_, thus improving the *J*_sc_. The low-index facets of the single-crystal nanostructure of the PtNCs provided the superb triiodide reduction ability to maximum electrocatalytic activity, which promoted the charge-transfer rate at the electrolyte–electrode interface and the exchange rate of the electrolyte and thus increased the *J*_sc_ and resulted a superior PCE.

**Table 4:** Summary of the photovoltaic characteristic parameters for the reference Pt film CE and the pulse-ECD PtNC CE

CE	*J*_sc_ (mAcm^−2^)	*V*_oc_ (*V*)	FF	*η* (%)
Pt film	15.18	0.70	0.58	6.18
PtNCs	15.33	0.69	0.61	6.48

## Conclusions

In this work, the single-crystal nanostructure of PtNCs was successfully developed at room temperature by the pulse-mode ECD technique to be deposited onto the FTO glass as a CE for DSSCs. Our results indicated that the crystallinity of PtNCs with a unique cubic shape exposed the low-index facets, which offered the superb ability for triiodide reduction and resulted in the superior electrocatalytic activity. The electrocatalytic activity of the prepared PtNC CE, as determined by the catalyst mass activity (MA) for the triiodide reduction reaction, was 2.71 times better than that of a conventional Pt film CE. Finally, the DSSCs assembled with the pulse-ECD PtNC CE showed a superior PCE of 6.48 % to the DSSCs with a conventional Pt film CE (6.18 %). PtNCs not only reduced the Pt loading to make a better Pt utilization efficiency but also improved the PCE of DSSCs. Present work suggests that the Pt CE with the outstanding electrocatalytic activities and low Pt loading could be achieved by controlling the crystallinity of Pt.
